# Sodium-glucose cotransporter 2 inhibitors—but not insulin—enhance renal branched-chain amino acid catabolism

**DOI:** 10.3389/fendo.2025.1706838

**Published:** 2025-11-07

**Authors:** Moeko Sakamoto, Nao Hasuzawa, Lixiang Wang, Kyoko Tashiro, Miyuki Kitamura, Shimpei Iwata, Nobuhiko Wada, Seiji Nomura, Sawako Moriyama, Mizuki Gobaru, Yukihiro Inoguchi, Ayako Nagayama, Kenji Ashida, Yoshinori Moriyama, Masatoshi Nomura

**Affiliations:** 1 Division of Endocrinology and Metabolism, Department of Internal Medicine, Kurume University School of Medicine, Kurume, Japan; 2 Department of Medical Biochemistry, Kurume University School of Medicine, Kurume, Japan; 3 Research Institute of Medical Mass Spectrometry, Kurume University School of Medicine, Kurume, Japan

**Keywords:** branched-chain amino acids (BCAA), SGLT2 inhibitors, type 2 diabetes, diabetic kidney disease, renal BCAA metabolism, urinary BCAA metabolites, insulin therapy

## Abstract

**Aims/hypothesis:**

Sodium–glucose cotransporter 2 inhibitors (SGLT2i) confer cardio-renal protection, and recent work implicates enhanced branched-chain amino acid (BCAA) catabolism as a potential mechanism in the heart. Whether SGLT2i also promotes renal BCAA catabolism is largely unknown. We hypothesized that SGLT2i enhances renal BCAA catabolism independently of glycemic effects.

**Methods:**

We conducted a prospective, single-center, open-label, nonrandomized, controlled clinical study in patients with type 2 diabetes stably treated with insulin, who were assigned to dapagliflozin (5 mg/day with dose-reduced insulin; n=8/9 completed) or insulin dose-up (n=5/8 completed). At 12 weeks, changes in urinary and plasma metabolites and short-chain acylcarnitines related to BCAA catabolism were quantified. To explore mechanisms, 10-week-old db/db mice received luseogliflozin (10 mg/kg/day, p.o.) or insulin glargine (10 U/day, s.c.) for 4 weeks; renal histology, mRNA and protein expression of key enzymes involved in BCAA catabolism, including branched-chain aminotransferase 2 (BCAT2), branched-chain ketoacid dehydrogenase (BCKDH), and BCKD kinase (BCKDK), were assessed.

**Results:**

Dapagliflozin treatment induced greater increases in urinary excretion of three BCAA-derived metabolites—3-hydroxypropionic acid, C5-OH carnitine, and 3-hydroxybutyric acid—compared with insulin at comparable glycemic levels. In contrast, C4 carnitine (an earlier metabolite in valine catabolism) rose more with insulin. No corresponding between-group differences were detected in plasma metabolites. In db/db mice, luseogliflozin attenuated glomerular mesangial expansion and tubular epithelial atrophy, and reduced Col1a1 mRNA and TGF-β1 protein, compared with glargine at comparable glycemic levels. Luseogliflozin decreased the phosphorylated (inactive) form of the BCKDH E1α subunit (p-BCKDHA/BCKDHA) and lowered BCKDK protein. mRNA expression of amino acid transporters and BCAT2 expression was unchanged.

**Conclusions/interpretation:**

Across complementary human and mouse studies, SGLT2 inhibition was suggested to enhance renal BCAA catabolism compared with insulin at comparable glycemic levels. In humans, increases in urinary BCAA-derived downstream metabolites without corresponding changes in plasma support a kidney-localized metabolic effect. In mice, SGLT2 inhibitor improved renal histopathology, and reduced phosphorylation-mediated inactivation of BCKDH. These findings provide mechanistic, translational evidence that SGLT2i modulate BCAA flux independently of glucose lowering, suggesting BCAA catabolism as a therapeutic axis in diabetic kidney disease.

**Clinical trial registration:**

https://rctportal.mhlw.go.jp/en/detail?trial_id=UMIN000052955, identifier UMIN000052955.

## 1 Introduction

Diabetic kidney disease (DKD) is a chronic complication of diabetes and a leading cause of end-stage renal disease (ESRD) (1). Studies have shown that as DKD progresses, both mortality rates and cardiovascular disease risk increase (2–4). The development of treatments to slow DKD progression remains a critical medical issue for improving patients’ quality of life and healthcare outcomes.

The advent of sodium-glucose co-transporter 2 (SGLT2) inhibitors has revolutionized DKD treatment. SGLT2 inhibitors lower blood glucose levels by inhibiting glucose reabsorption in renal proximal tubule cells (5). SGLT2 inhibitors are reported to significantly reduce all-cause mortality, cardiovascular mortality, hospitalization due to heart failure, and ESRD risk (6–8).

Several hypotheses have been proposed to explain the cardiorenal protective mechanisms of SGLT2 inhibitors. These drugs promote weight loss, primarily by reducing fat mass (9, 10), enhance insulin sensitivity, and lower blood pressure and serum uric acid levels (11). Furthermore, SGLT2 inhibitors increase circulating levels of ketone bodies and enhance their utilization, improving oxygen consumption efficiency (12). Ketone bodies also possess anti-inflammatory properties (13). While these mechanisms partially explain the clinical cardiorenal benefits, further research is needed to fully elucidate the effects of SGLT2 inhibitors.

Recently, activation of branched-chain amino acids (BCAA) catabolism in the heart has emerged as a potential mechanism for the cardioprotective effects of SGLT2 inhibitors. Empagliflozin has been shown to ameliorate adverse cardiac remodeling and heart failure in a non-diabetic porcine model by shifting myocardial fuel utilization towards BCAA (14). Although kidneys also have high BCAA oxidation flux (15), few studies have investigated renal BCAA catabolism in this context.

Previously, Sharma et al. identified 13 metabolites that were significantly decreased in patients with diabetes and CKD compared with healthy controls and/or patients with diabetes without CKD (16). We noted that six metabolites among these—3-hydroxypropionic acid, 3-methylcrotonylglycine, 2-methylacetoacetate, 3-hydroxyisovalerate, 2-ethyl-3-hydroxypropionate, and tiglylglycine—are all products of branched-chain amino acid (BCAA) catabolism. Furthermore, a study conducted by Mulder et al. demonstrated that treatment with dapagliflozin increased the levels of the first three of these six metabolites (3-hydroxypropionic acid, 3-methylcrotonylglycine, and 2-methylacetoacetate) compared with pre-intervention levels (17). Based on these findings, we hypothesized that SGLT2 inhibitors may enhance renal BCAA catabolism, which is impaired in DKD.

BCAA catabolism occurs mainly within mitochondria. In the first step, BCAAs are converted to branched-chain α-ketoacids (BCKAs) by mitochondrial branched-chain aminotransferase 2 (BCAT2). During this process, nitrogen from BCAAs is transferred to α-ketoglutarate, forming glutamate. Because hepatic BCAT2 activity is very low, this first step mainly occurs in other organs, including the kidneys and skeletal muscle (15). In the second step, BCKAs are converted into branched-chain acyl-CoA (BCA-CoA) by branched-chain α-ketoacid dehydrogenase complex (BCKDC). This step is irreversible and is the rate-limiting step in BCAA breakdown. BCKDC is inactivated by another enzyme, branched-chain α-ketoacid dehydrogenase kinase (BCKDK), which is activated in insulin resistance (18). Elevated plasma BCAA levels are associated with insulin resistance (19). Elucidating how SGLT2 inhibitors affect renal BCAA metabolism is an important step forward in understanding their glucose-independent renal protective effects and for identifying potential therapeutic pathways.

This study analyzed the effects of SGLT2 inhibitor administration on renal BCAA metabolism in patients with type 2 diabetes and in db/db mice, which have been reported to recapitulate the elevations in plasma BCAAs observed in humans with type 2 diabetes (15). To distinguish drug-specific effects of SGLT2 inhibitors from general changes induced by glucose-lowering, we compared the effects of SGLT2 inhibitors to those of insulin, maintaining comparable blood glucose levels between groups.

## 2 Materials and methods

### 2.1 Patient enrollment

We conducted a prospective, single-center, open-label, nonrandomized, controlled trial at Kurume University Hospital (Kurume, Japan) from January 2020 to October 2022. Inclusion criteria comprised a diagnosis of type 2 diabetes with HbA1c > 47 mmol/mol (6.5%), age ≥20 and <80 years, a stable antidiabetic treatment with insulin for at least 8 weeks prior to the study, and no prior use of SGLT2 inhibitors. Exclusion criteria encompassed pregnant or breastfeeding women and patients with malignant disease, severe infections (including urinary tract infections), severe trauma, or recent surgery. After baseline examinations, patients were divided into two groups: the dapagliflozin group received 5 mg/day of dapagliflozin in addition to a reduced insulin dose (approximately 80% of baseline), while the insulin group received an increased insulin dose. In each group, clinical data were evaluated at baseline and 12 weeks after initiating treatment. This study was approved by the ethics committee of Kurume University, and the protocol complied with the Declaration of Helsinki. All patients provided their written informed consent before enrollment. The study was registered with UMIN Clinical Trials Registry (UMIN000052955).

### 2.2 Study endpoints

The study endpoints included changes in plasma and urinary amino acids and metabolites, anthropometric parameters, and blood parameters. Blood parameters included HbA1c, fasting plasma glucose (FPG), serum C-peptide (CPR), LDL and HDL cholesterol, triglycerides (TG), alanine aminotransferase (ALT), aspartate aminotransferase (AST), blood urea nitrogen (BUN), creatinine (Cr), uric acid (UA), 3-hydroxybutyric acid, liver-type fatty acid-binding protein (L-FABP), and urinary albumin-to-creatinine ratio (mg/g). All parameters were measured after an overnight fasting period of at least 8 hours.

### 2.3 Animals

We used male mice exclusively to avoid potential confounding effects of estrogen on renal injury development. Db/db male mice (BKS.Cg-Dock7m+/+LeprdbJ) and db/+ mice were obtained from Charles River Laboratories Japan, Inc. The mice were housed individually and provided ad libitum access to diet (D12450JM, Research Diets, New Brunswick, NJ, USA) and were maintained under a 12-h light/dark cycle. Ten-week-old male db/db mice were randomly assigned to experimental groups (n = 4–8 per group) and subjected to the intervention for 4 weeks, while an untreated control group was maintained under the same conditions. The luseogliflozin-treated group received a daily oral gavage of 10 mg/kg luseogliflozin diluted in water, while the glargine-treated group received a daily subcutaneous injection of 10 U insulin glargine. The doses of drugs were determined based on previous studies (20–22). Random blood glucose levels were measured weekly using a Glutest Sensor (Sanwa Kagaku, Nagoya, Japan). Mice were maintained under ad libitum feeding conditions and sacrificed under isoflurane anesthesia at Zeitgeber time 6 (mid-light phase) for tissue collection. Histological analyses were performed in four groups: db/db control, db/db luseogliflozin, db/db glargine, and db/+ as a non-diabetic control. The db/+ group was excluded from other quantitative analyses to account for the potential effects of genetic background. All experiments were conducted in accordance with institutional guidelines and approved by the ethics committee of Kurume University School of Medicine (approval number: 2020-131).

### 2.4 Clinical analysis

Amino acids and metabolites in urine and plasma samples from patients were analyzed by HPLC or GC/MS. Other laboratory analyses were performed by the hospital laboratory or SRL Inc (Tokyo, Japan). Body weight, BMI, skeletal muscle weight, and skeletal muscle percentage were measured using a body composition analyzer (LookingBody 120, Tokyo, Japan) employing the bioimpedance method. eGFR was calculated using revised equations for Japanese individuals.

### 2.5 Blood and urine biochemistry

Amino acids in urine and plasma samples from patients were analyzed by HPLC. HPLC was performed using JLC-500/V fully automatic high-speed amino acid analyzer (JEOL, Tokyo, Japan) according to the manufacturer’s procedures. Briefly, the urine and plasma samples were deproteinized with 3% sulfosalicylic acid, and the supernatants were filtered through a 0.45 µm filter. These samples were analyzed by an automated amino acid analyzer consisting of cation exchange chromatography and post-column ninhydrin detection.

Metabolites in urine and plasma of patients were measured by gas chromatography-mass spectrometry (GC/MS) as described previously (23, 24). Each analyte was identified from the spectrum, and the area under the spectrum of targeted metabolites was divided by that of heptadecanoic acid to acquire the ratio of each metabolite, which was further normalized to creatinine.

Acylcarnitines in urine and plasma of patients were measured by high-performance liquid chromatography–tandem mass spectrometry (HPLC-MS/MS) as described previously (25). Each analyte was identified from the spectrum, and the area under the spectrum of each targeted metabolite was standardized using stable isotope standards of the respective carnitines at known concentrations to determine metabolite concentration. The urinary concentration was further normalized to urinary creatinine.

### 2.6 Histopathological analysis

Mouse kidney samples were fixed in 4% paraformaldehyde, embedded in paraffin, sectioned, and stained with hematoxylin-eosin or periodic acid-Schiff (PAS). Glomerular and mesangial areas were quantified by analyzing 10 randomly selected glomeruli per animal, and the mean value for each animal was used for statistical analysis (n = 4 per group). Immunostaining was performed on paraffin-embedded sections using a pS6 monoclonal antibody (1:200) or a cystatin C antibody (1:200). For fluorescence quantification, images were randomly captured by a blind investigator. Images were analyzed using BZ-X Hybrid Cell Count software (Keyence).

### 2.7 mRNA analysis

Total RNA was extracted from mouse kidney samples using TRIzol reagent (Invitrogen). Reverse transcription was carried out using 1 μg of RNA and the QuantiTect Reverse Transcription Kit (Qiagen). Quantitative real-time PCR was performed using TB Green Premix Ex Taq II (Takara Bio) on the Step-One plus Real-Time PCR System (Applied Biosystems). The primer sequences for the selected genes are listed in the **Supplementary Table S1**. Results were normalized to *Gapdh* expression and presented as fold changes relative to gene expression in the control mice.

### 2.8 Western blot analysis

Mouse kidney homogenates were prepared using Western lysis buffer (20 mM Tris-HCl pH 7.6, 150mM NaCl, 2 mM EDTA, 0.5% NP-40) containing protease and phosphatase inhibitor tablets (Roche Applied Science, Mannheim, Germany). Protein levels in the samples were quantified using a bicinchoninic acid (BCA) protein assay kit (Thermo Fisher Scientific), then mixed with Laemmli sample buffer (Bio-Rad, Hercules, CA, USA) and heated at 95 °C for 5 min. A total of 10 μg of protein was separated by SDS-PAGE and transferred onto a PVDF membrane. After blocking, the blots were incubated overnight at 4 °C with primary antibodies (1:1000) against pS6 (Ser235/236), tS6, p-p70 S6 Kinase (Thr389; pS6K), p70 S6 Kinase (S6K), eEF2K, KLF15, BCAT2, p-BCKDHA(S293), BCKDHA, PPM1K, or BCKDK; primary antibodies (1:2000) against fibronectin, eEF2, or DLD; or a primary antibody against TGF-β1 (1:500). Horseradish peroxidase (HRP)-conjugated secondary antibodies (1:2000) were applied for 1h at room temperature, followed by chemiluminescence detection using the Western Blotting Detection System (GE Healthcare, Buckinghamshire, UK). Relative band intensities were quantified using ImageJ software (NIH, Bethesda, MD, USA). The antibodies used are listed in **Supplementary Table S2**. Full blot images are presented in **Supplementary Figures S3-S5**.

### 2.9 Statistical analyses

Statistical analyses were performed using GraphPad Prism 10 software (GraphPad, San Diego, CA, USA). Sample sizes are reported in the results section and figure legends. For the comparison of baseline characteristics, including sex and concomitant medication use, Fisher’s exact test was used. The Shapiro–Wilk test was used to assess the normality of the data. Clinical trial data were reported as the mean ± SD or the median (interquartile range), depending on the distribution. For normally distributed data, differences were analyzed using an unpaired two-tailed Student’s t-test. For non-normally distributed data, differences were analyzed using the Mann–Whitney test. For animal studies, results are presented as the mean ± SEM. Statistical significance among three or more groups was determined using two-way ANOVA followed by Tukey’s or Dunnett’s *post hoc* test. P-values <0.05 were considered statistically significant.

## 3 Results

### 3.1 Dapagliflozin treatment increases urinary excretion of BCAA metabolites compared with insulin in patients with diabetes

**Figure 1:**
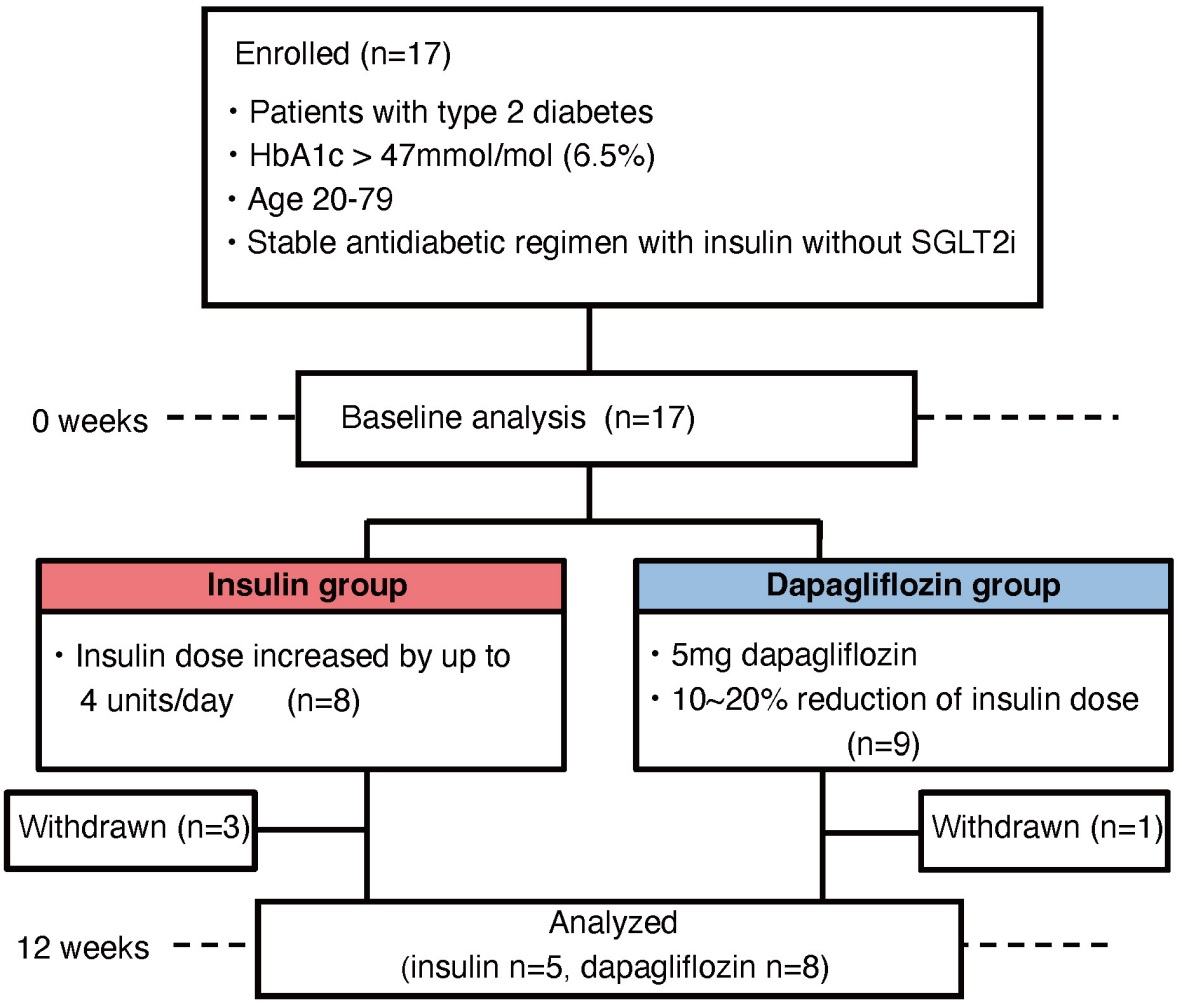
Flowchart of patient enrollment.

Seventeen patients were enrolled in the study, eight in the insulin group and nine in the dapagliflozin group (**Figure 1**). Baseline characteristics including the use of concomitant medications were similar between the two groups (**Table 1**). During the study, three patients in the insulin group withdrew due to development of malignant disease (n=2) or heart failure (n=1), while one patient in the dapagliflozin group withdrew due to an adverse event (increased urine output). Five patients in the insulin group and eight in the dapagliflozin group completed the 12-week intervention. No participants experienced or reported symptomatic hypoglycemia.

**Table 1:** Baseline characteristics of the patients.

Characteristic	Insulin group	Dapagliflozin group	P-value
N (female/male)	5 (3/2)	8 (2/6)	0.293
Age (years)	72.2 ± 2.9	62.0 ± 9.9	0.084
Weight (kg)	66.8 ± 16.7	62.2 ± 9.6	0.787
Body mass index (kg/m²)	24.9 ± 5.5	25.2 ± 5.9	0.877
FPG (mg/dL)	142.2 ± 28.2	151.1 ± 33.4	0.656
HbA1c (mmol/mol)	58.6 ± 6.8	57.9 ± 7.8	0.872
HbA1c (%)	7.6 ± 0.6	7.4 ± 0.6	0.908
Estimated GFR (mL/min/1.73 m²)	59.3 ± 25.3	78.2 ± 14.8	0.145
UACR (mg/g)	35 (5.3-109.1)	22.8 (16.3-78.3)	0.943
Medication, n (%)			
Sulfonylurea	0 (0)	0 (0)	>0.999
Glinides	1 (20)	0 (0)	0.385
DPP-4 inhibitor	2 (40)	2 (25)	>0.999
Metformin	1 (20)	3 (37.5)	>0.999
Pioglitazone	0 (0)	0 (0)	>0.999
α-Glucosidase inhibitor	1 (20)	0 (0)	0.385
GLP-1RA	1 (20)	1 (12.5)	>0.999
SGLT2 inhibitor	0 (0)	0 (0)	>0.999
Insulin	5 (100)	8 (100)	>0.999

Baseline characteristics of clinical study patients. N indicates the number of patients, reported as female/male based on self-reported sex. Data are presented as mean ± SD or medians (interquartile range), depending on the distribution. Statistical analysis was performed using unpaired Student’s *t-*test, the Mann–Whitney test or Fisher’s exact test. *p<0.05.

FPG, fasting plasma glucose; UACR, urine albumin-to-creatinine ratio.

The 12-week intervention did not induce significant changes in glycemic control in either group (**Table 2**), likely due to the limited increase in insulin dose (2–4 units/day) in the insulin group and the reduction of insulin dose (approximately –20%) in the dapagliflozin group. There were no significant changes in body weight, skeletal muscle mass, skeletal muscle percentage, or body fat in the insulin group. In contrast, the dapagliflozin group showed a non-significant trend toward decreased body weight and a significant decrease in body fat mass and percentage (**Table 2**). Skeletal muscle mass was preserved in both groups, while the skeletal muscle percentage was significantly increased in the dapagliflozin group, reflecting the reduction in body fat. However, this observation should be interpreted with caution because of the unequal distribution of sex and age between the groups (**Table 1**). In the insulin group, eGFR increased modestly, whereas it remained stable with dapagliflozin. Given that renal function markers, including urinary albumin and L-FABP, did not show significant changes in either group, the slight eGFR increase in the insulin group might reflect hemodynamic variation.

**Table 2:** Clinical parameters at baseline and after 12 weeks of treatment.

Parameter	Insulin group			Dapagliflozin group		
	Baseline	12 weeks	p-value	Baseline	12 weeks	p-value
Body weight (kg)	62.4 ± 16.4	63.1 ± 17.2	0.263	62.2 ± 9.6	60.7 ± 9.2	0.096
Body mass index (kg/m²)	23.9 ± 5.5	24.2 ± 5.4	0.295	23.8 ± 2.7	23.3 ± 2.7	0.100
Skeletal muscle mass (kg)	23.3 ± 4.6	23.4 ± 4.6	0.474	23.5 ± 4.5	23.9 ± 4.8	0.132
Skeletal muscle percentage (%)	36.7 ± 4.1	37.8 ± 4.2	0.609	36.7 ± 3.9	39.2 ± 4.5	0.023*
Body fat amount (kg)	19.0 ± 9.9	19.5 ± 10.6	0.372	18.8 ± 4.6	16.7 ± 4.6	0.024*
Body fat percentage (%)	29.0 ± 7.7	29.0 ± 8.0	0.684	30.1 ± 6.5	27.1 ± 7.5	0.025*
FPG (mg/dL)	142.2 ± 28.2	151.8 ± 52.5	0.546	151.1 ± 33.4	132.4 ± 25.4	0.185
HbA1c (mmol/mol)	58.6 ± 6.8	60.8 ± 13.1	0.851	57.9 ± 7.8	56.6 ± 7.3	0.745
HbA1c (%)	7.6 ± 0.6	7.7 ± 1.2	0.595	7.5 ± 0.7	7.4 ± 0.6	0.541
AST (U/L)	16 (16–19)	17 (17–17)	0.813	20 (15.8-24.3)	19.5 (15.8-23.3)	0.422
ALT (U/L)	16.4 ± 8.0	14.0 ± 4.6	0.329	22.5 ± 11.9	20.4 ± 10.4	0.164
γ-GTP (U/L)	18.0 ± 7.1	17.6 ± 8.1	0.799	21.0 ± 9.6	18 ± 6.3	0.138
LDL-C (mg/dL)	105.2 ± 9.2	103.6 ± 11.1	0.866	109.8 ± 31.6	108.6 ± 36.1	0.834
HDL-C (mg/dL)	53.4 ± 9.6	52.2 ± 12.0	0.762	43.5 ± 9.4	46.1 ± 9.6	0.243
TG (mg/dL)	108.2 ± 35.4	114.2 ± 47.0	0.664	105 (72.8-154.8)	76 (68.3-211.5)	0.844
BUN (mg/dL)	20.4 ± 7.5	18.6 ± 5.0	0.532	16.6 ± 2.4	18.0 ± 3.7	0.211
Cr (mg/dL)	1.01 ± 0.5	0.9 ± 0.5	0.030*	0.8 ± 0.2	0.7 ± 0.1	0.612
eGFR (ml/min/1.73m²)	59.3 ± 25.3	63.6 ± 25.8	0.007*	78.2 ± 14.8	78.6 ± 12.1	0.899
UA (mg/dL)	5.8 ± 0.8	5.3 ± 0.7	0.043*	5.7 ± 1.0	5.3 ± 0.8	0.070
UACR (mg/g)	35.0 (5.3-109.1)	21.4 (13–0239.1)	0.313	22.8 (16.3-78.3)	34.6 (19.1-60.0)	0.844
L-FABP (ng/ml)	4.3 ± 3.4	6.4 (2.6-10.9)	0.313	3.5 (2.8-7.0)	2.9 (2.5-5.3)	0.641

Clinical parameters of study patients before and after intervention with dapagliflozin or insulin dose-up treatment. Data are presented as mean ± SD or median (interquartile range), based on distribution. Statistical analysis was performed using unpaired Student’s *t-*test or the Mann–Whitney test, respectively. *p<0.05.

FPG, fasting plasma glucose; AST, aspartate aminotransferase; ALT, alanine aminotransferase; γ-GTP, γ-glutamyl transpeptidase; TG, triglycerides; BUN, blood urea nitrogen; Cr, creatinine; UA, uric acid; UACR, urine albumin-to-creatinine ratio; L-FABP, liver-type fatty acid-binding protein.

**Figure 2:**
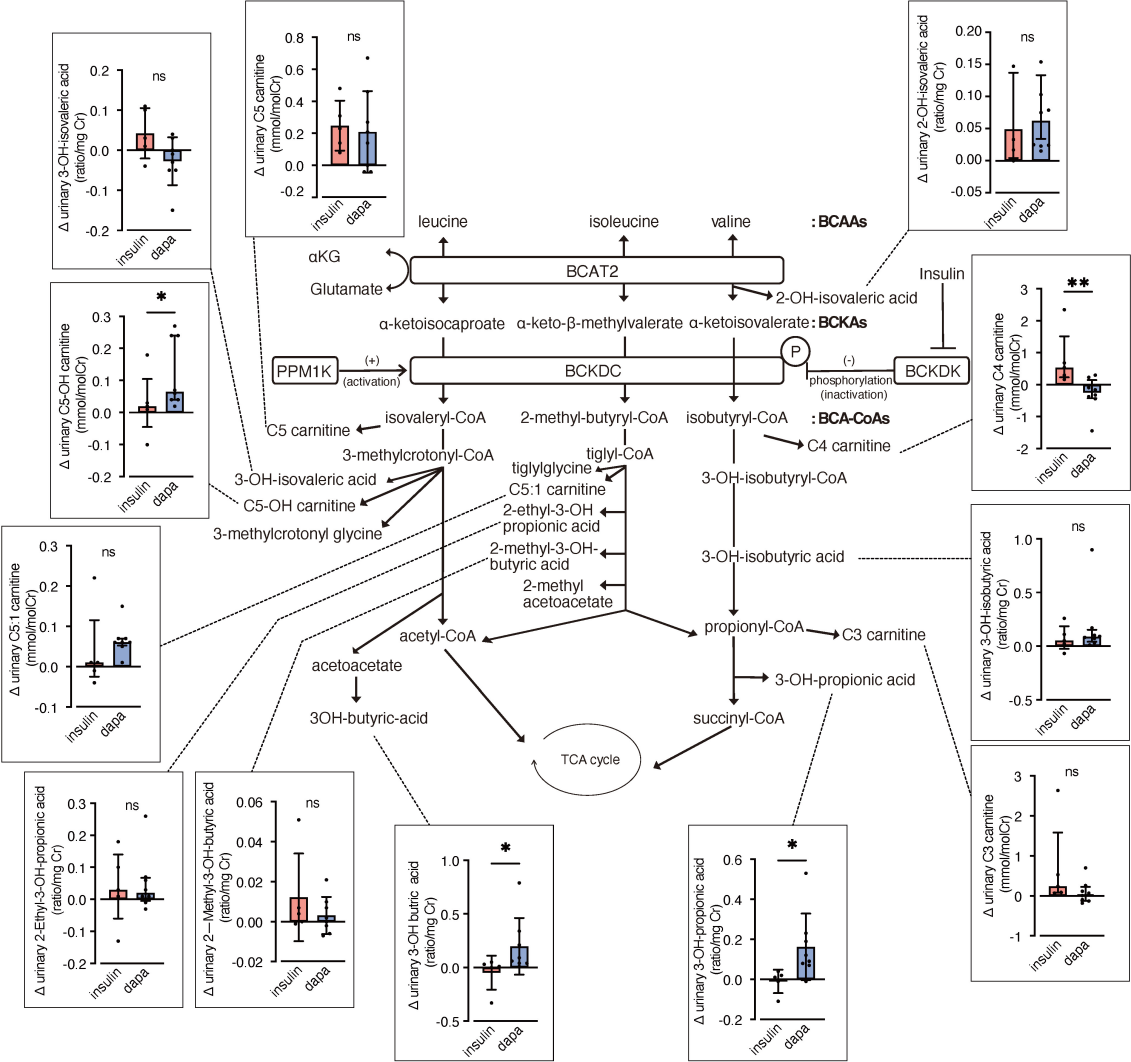
Changes in urinary BCAA metabolites induced by dapagliflozin or insulin dose-up treatment. The BCAA catabolic pathway and changes in the levels of urinary metabolites and acylcarnitines related to BCAA catabolism after 12 weeks of insulin dose-up or dapagliflozin treatment. Data are expressed as mean ± SD for normally distributed variables, or as median with error bars representing the interquartile range for non-normally distributed variables. Statistical analysis was performed using unpaired Student’s *t-*test or the Mann–Whitney test, respectively. *p<0.05. TCA cycle, Tricarboxylic acid cycle; αKG, α-ketoglutarate; BCAA, branched chain amino acid; BCKA, branched-chain α-ketoacids; BCA-CoA, branched-chain acyl-CoA; BCAT2, branched-chain aminotransferase 2; BCKDC, branched-chain α-ketoacid dehydrogenase complex; BCKDK, branched-chain α-ketoacid dehydrogenase kinase; PPM1K, protein phosphatase, Mg2+/Mn2+ dependent 1K.

**Figure 3:**
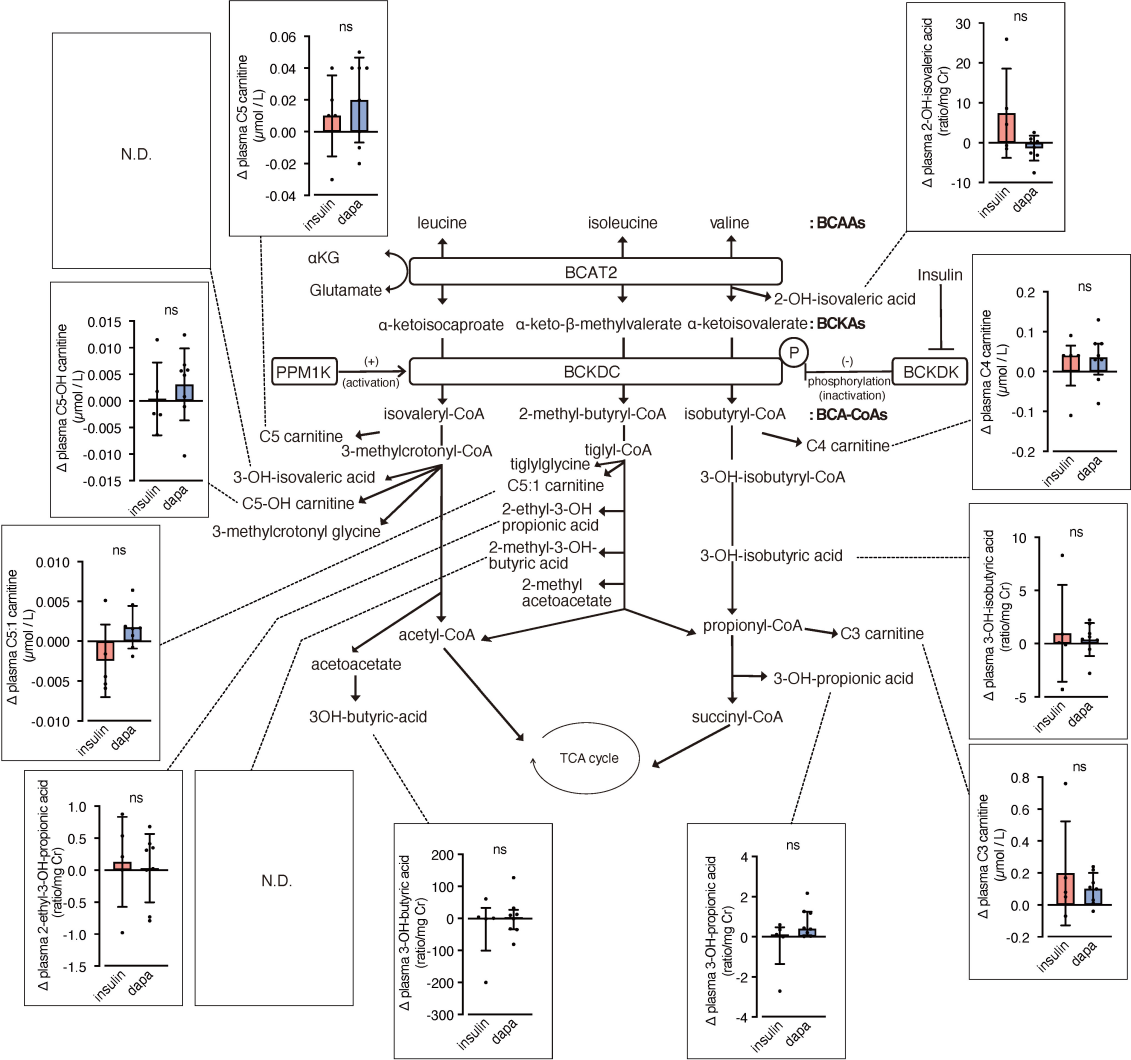
Changes in plasma BCAA metabolites induced by dapagliflozin or insulin dose-up treatment. The BCAA catabolic pathway and changes in the levels of plasma metabolites and acylcarnitines related to BCAA catabolism after 12 weeks of insulin dose-up or dapagliflozin treatment. Data are expressed as mean ± SD for normally distributed variables, or as median with error bars representing the interquartile range for non-normally distributed variables. Statistical analysis was performed using unpaired Student’s *t-*test or the Mann–Whitney test, respectively. *p<0.05..

We analyzed urinary levels of metabolites and short-chain acylcarnitines (C3, C4, and C5 carnitine) related to BCAA catabolism, including six metabolites previously identified as DKD biomarkers. Because absolute values varied considerably among patients, we analyzed changes from before to after the intervention. BCAA downstream metabolites—C5-OH carnitine [a leucine-derived metabolite], 3-hydroxypropionic acid [an isoleucine and valine-derived metabolite], and 3-hydroxybutyric acid [also known as a leucine-derived metabolite]—exhibited significantly greater increases in the dapagliflozin group compared with the insulin group (**Figure 2**). These findings are consistent with those of Mulder et al., who reported increases in 3-hydroxypropionic acid and two other metabolites, 3-methylcrotonylglycine and 2-methylacetoacetate, which were not detected in the present assay, after dapagliflozin treatment. In contrast, C4 carnitine, an earlier metabolite in valine catabolism, showed a significantly greater increase in the insulin group compared with the dapagliflozin group. Other DKD biomarker metabolites identified by Sharma et al., as well as the DKD metabolite score (MSDKD) calculated based on Mulder et al. (17), showed no significant differences in changes between treatment groups (**Supplementary Figures S1A, B**). We also measured these BCAA metabolites in plasma; however, no significant between-group differences in changes corresponding to those observed in urine were detected (**Figure 3**). Similarly, no significant group differences were observed in plasma concentrations of other DKD biomarker metabolites (**Supplementary Figure S1C**).

### 3.2 Dapagliflozin does not significantly affect blood levels of branched-chain amino acids in patients with diabetes

**Figure 4:**
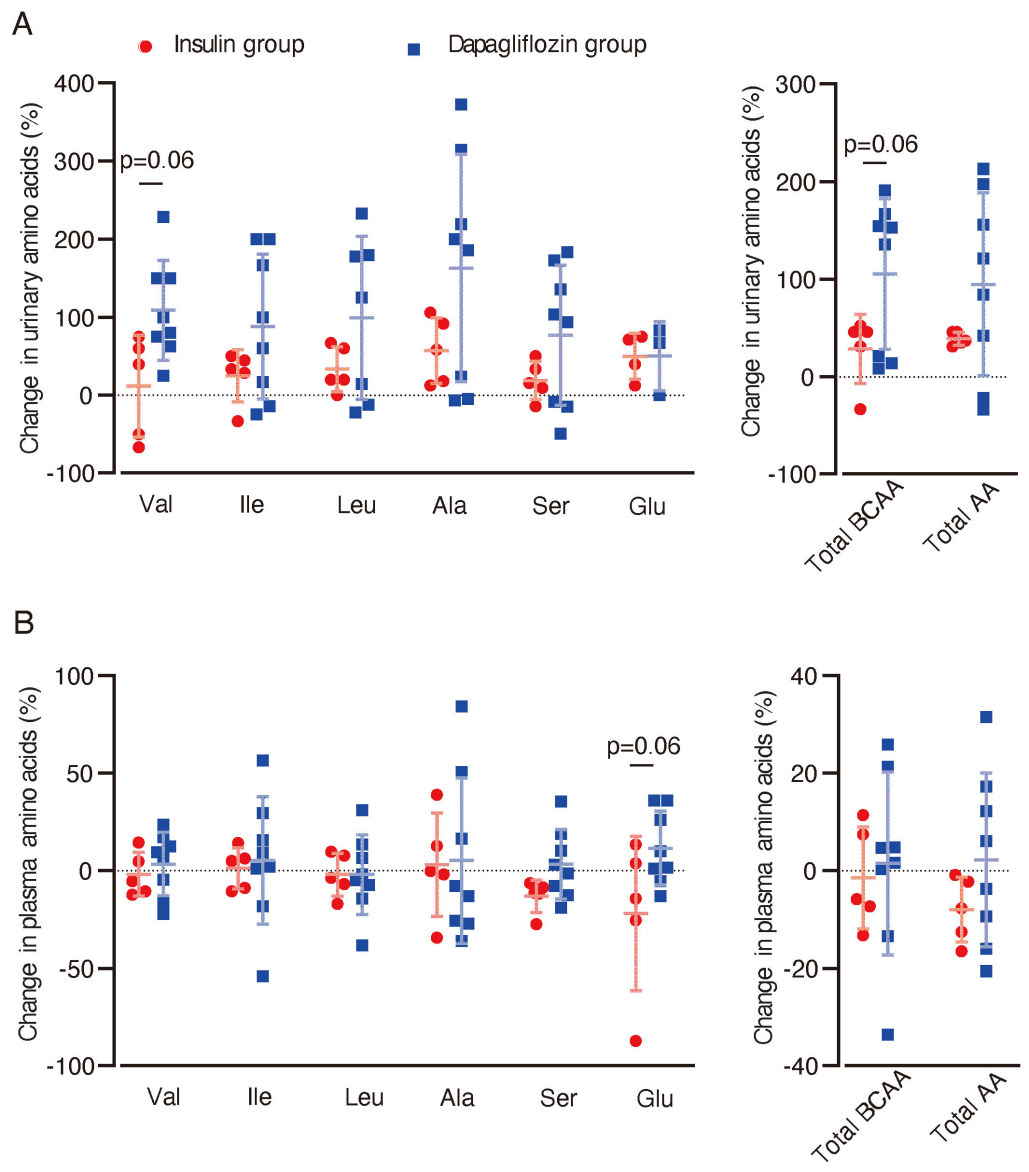
Changes in urinary and plasma amino acid concentrations after dapagliflozin or insulin dose-up treatment. Changes in urinary and plasma amino acid concentrations between baseline and 12 weeks after the intervention were analyzed. **(A)** Percentage changes (%) from baseline in urinary levels of amino acids after 12 weeks of intervention. **(B)** Percentage changes (%) in plasma levels of amino acids from baseline (dapagliflozin *n=*8, insulin *n=*5). Data are expressed as mean ± SD. Statistical analysis was performed using unpaired Student’s *t-*test. Val, valine. Ileu, isoleucine; Leu, leucine; Ala, alanine; Ser, serine; Glu, glutamic acid.

To assess the potential effect of dapagliflozin on BCAA metabolism in the kidney, we analyzed amino acid concentrations in urine and blood. In urine samples, there was a trend towards increased levels of valine (p = 0.06) and total BCAA (p = 0.06) in the dapagliflozin group compared with the insulin group (**Figure 4A**). No significant changes or trends in plasma BCAA levels were observed between the two groups (**Figure 4B**). In contrast, blood glutamate (Glu) levels showed a trend towards an increase in the dapagliflozin group compared with the insulin group (p = 0.06).

### 3.3 Luseogliflozin reduces mesangial expansion and tubular epithelial atrophy compared with insulin glargine in db/db mice

**Figure 5:**
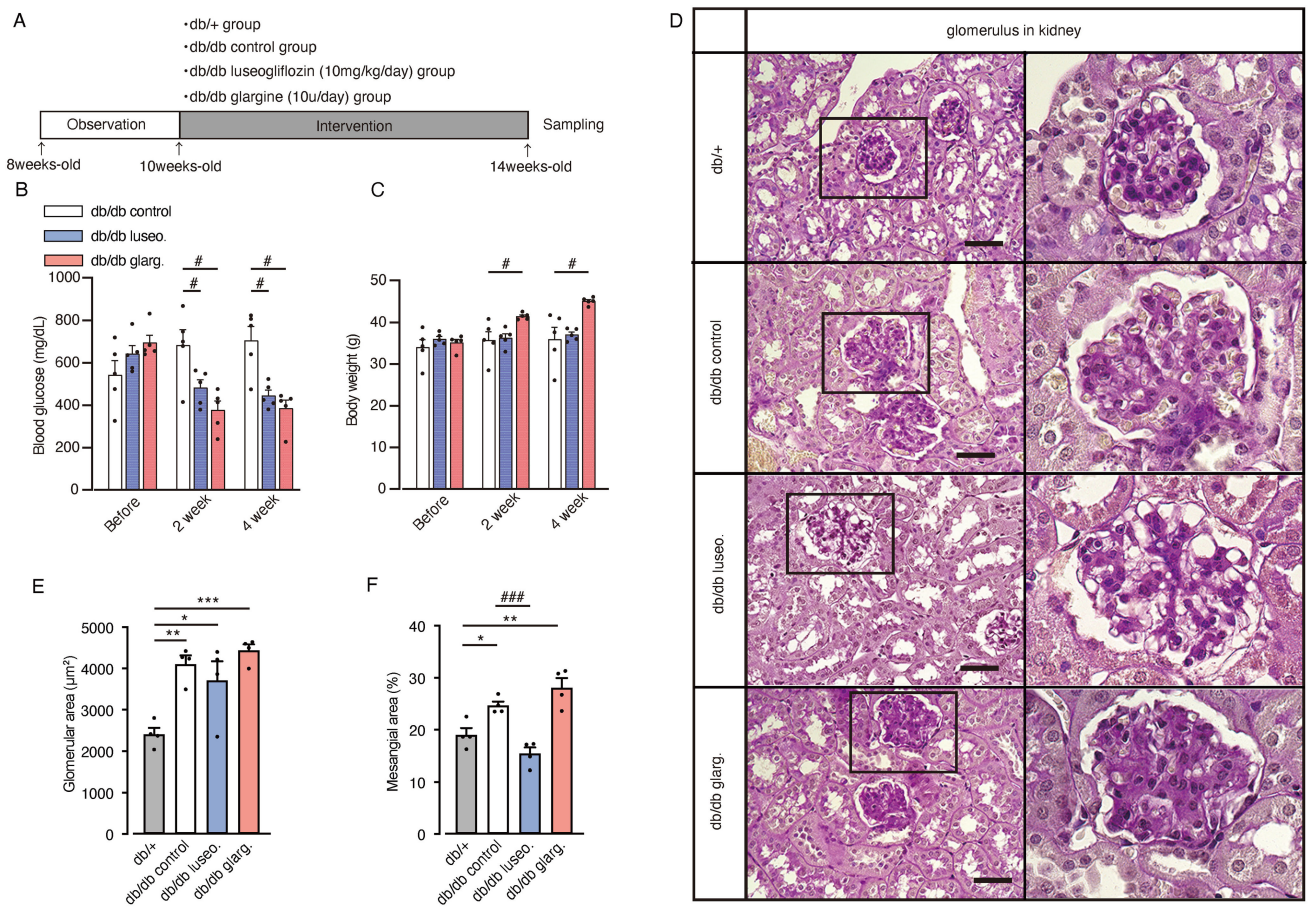
Effect of luseogliflozin and insulin glargine treatment on glomeruli of db/db diabetic mice. Db/db diabetic mice were treated with luseogliflozin (10 mg/kg/day, oral) or insulin glargine (10 U/body/day, s.c.). **(A)** Workflow of the animal experiment. **(B, C)** Blood glucose levels **(B)** and body weight **(C)** of mice before and after the intervention. **(D)** Representative PAS staining of kidney sections after 4 weeks of intervention. **(E, F)** Glomerular cross-sectional area (μm²) **(E)** and percentage of PAS-positive mesangial area (%) **(F)** were quantified in 10 randomly selected glomeruli per animal. Data are expressed as mean ± SEM of 4–5 mice per group. Statistical analysis was performed using two-way ANOVA followed by Tukey’s or Dunnett’s *post hoc* test. Scale bar, 50 μm. *p<0.05 versus db/+, # *p*<0.05 versus db/db control. PAS staining, periodic acid-Schiff staining.

To further investigate the effects of SGLT2 inhibitors on renal branched-chain amino acid (BCAA) metabolism, we performed analyses in diabetic db/db mice. Ten-week-old db/db mice were subjected to a 4-week treatment regimen with luseogliflozin (10 mg/kg/day, oral) or insulin glargine (10 U/day, s.c.), while other db/db mice and db/+ mice served as untreated and non-diabetic controls, respectively (**Figure 5A**). Both luseogliflozin and glargine treatment significantly reduced blood glucose levels compared with the control group (**Figure 5B**). Glargine treatment led to significant body weight gain, whereas luseogliflozin did not cause weight changes compared with the control group (**Figure 5C**).

Histological analysis revealed glomerular hypertrophy in all groups (control, luseogliflozin-treated, and insulin-treated), compared with non-diabetic db/+ mice (**Figures 5D, E**). Luseogliflozin significantly reduced mesangial expansion in db/db mice to levels similar to those in non-diabetic db/+ mice. This effect was not observed with glargine (**Figures 5D, F**).

**Figure 6:**
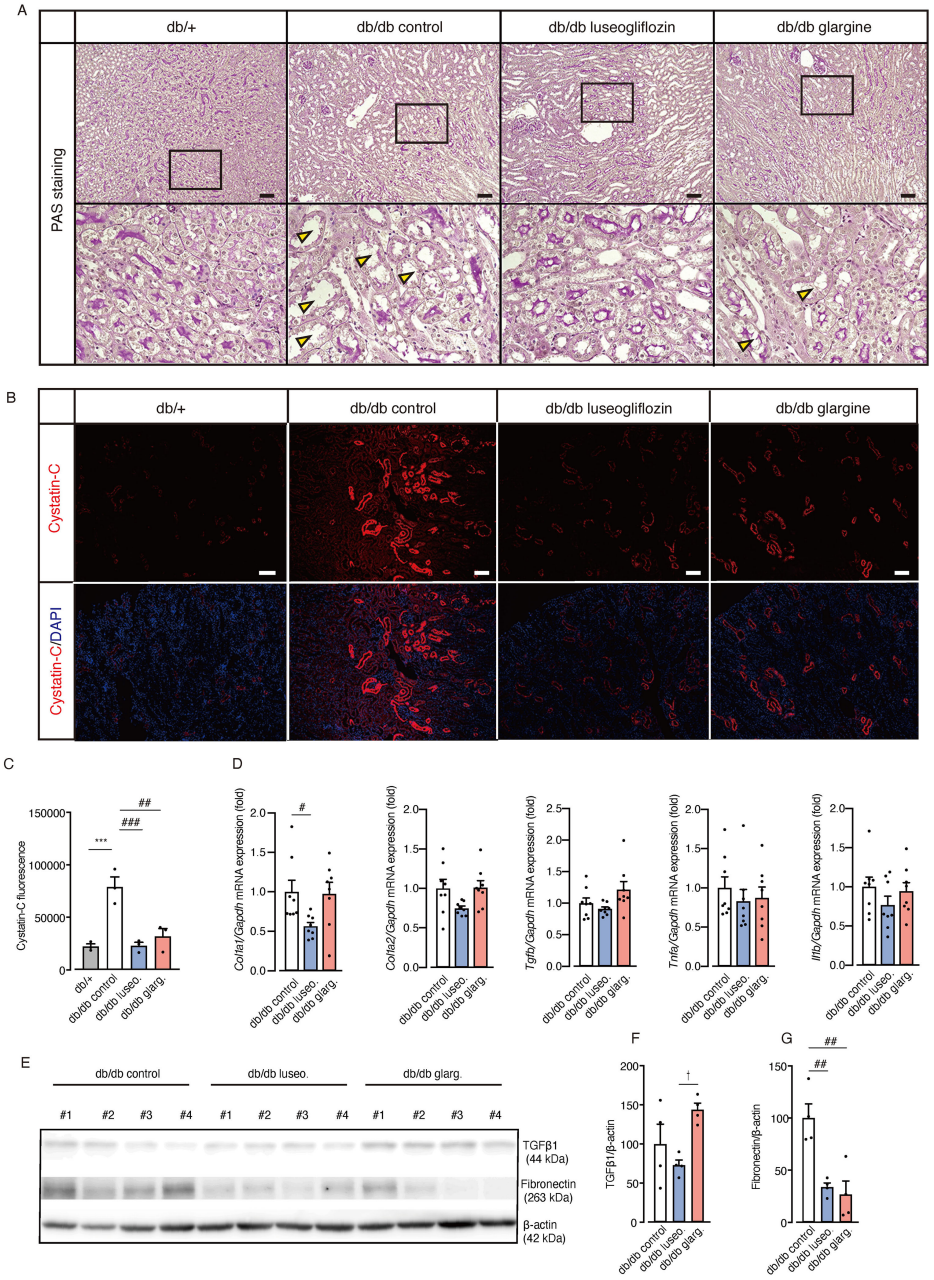
Effect of luseogliflozin and insulin glargine treatment on tubular epithelial atrophy, renal injury, and fibrosis in db/db diabetic mice. **(A)** Representative PAS staining of renal tubules after 4 weeks of intervention. Arrowheads indicate tubular atrophy with brush border loss. **(B, C)** Immunofluorescence staining for the proximal tubule injury marker cystatin-C **(B)** and quantitative analysis of fluorescence intensity **(C)**. **(D)** Kidney mRNA expressions of *Col1a1, Col1a2*, *Tgf-β1, Tnf-α, and Il-1β*. **(E-G)**. Western blot analysis and quantification of TGFβ1, Fibronectin, and β-actin in kidney lysates. Data are mean ± SEM of 3–8 mice per group. Statistical analysis was performed using two-way ANOVA followed by Tukey’s or Dunnett’s *post hoc* test. Scale bar, 100 μm. * p<0.05 versus db/+, †p < 0.05 db/db luseogliflozin versus db/db glargine. # p<0.05 versus db/db control. *Col Ia1, Ia2*, collagen Ia1, Ia2. PAS staining, periodic acid-Schiff staining.

Moreover, db/db control mice exhibited prominent tubular epithelial atrophy, reduced PAS staining in the brush border, and thickening of the basement membranes compared with non-diabetic db/+ mice (**Figure 6A**). Notably, luseogliflozin, but not glargine, reversed these pathological changes. Immunostaining revealed increased expression of the tubular injury marker cystatin-C in db/db mice compared with non-diabetic db/+ mice (**Figures 6B, C**). Both luseogliflozin and glargine treatment reversed the increase in cystatin-C expression (**Figure 6C**). *Collagen type I alpha 1 (Col1a1)* mRNA expression, an indicator of fibrosis, was significantly reduced in luseogliflozin-treated mice compared with the control group, whereas no significant reduction was observed in the glargine-treated mice (**Figure 6D**). *Tgfb, Tnfa*, and *Il1b* mRNA expression levels showed no significant differences between the groups. Western blot analysis of fibrosis-related factors revealed a significant reduction in TGF-β1 expression in luseogliflozin-treated mice compared with the glargine group, consistent with the changes in *Col1a1* mRNA expression, one of the target genes of TGF-β (**Figures 6E, F**). Fibronectin expression was significantly reduced in the kidney tissue from both luseogliflozin- and glargine-treated groups compared with the control group (**Figures 6E, G**).

**Figure 7:**
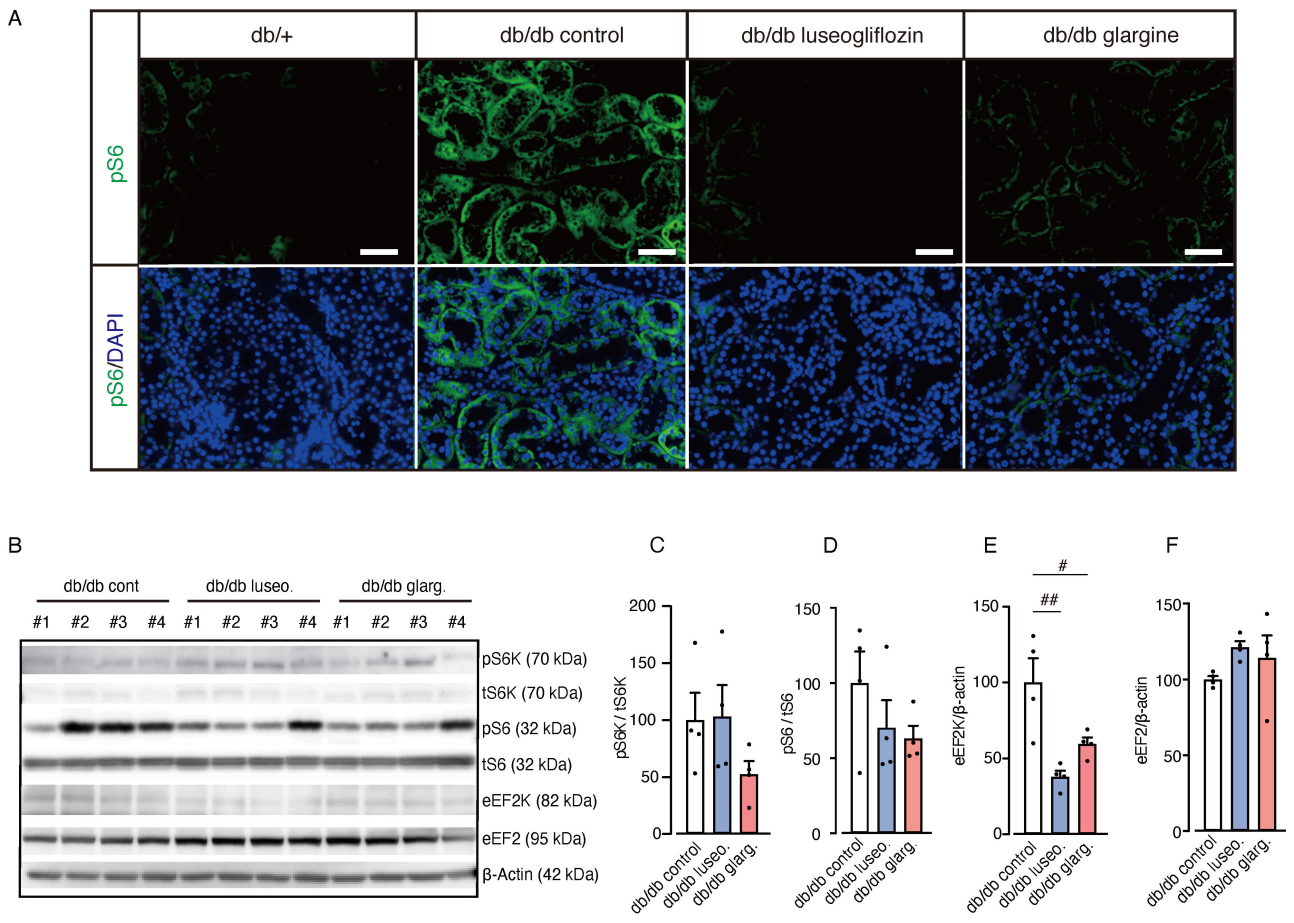
Effect of luseogliflozin and insulin glargine treatment on mTORC1 activity in db/db mice. The effect of luseogliflozin and insulin glargine treatment on renal mTORC1 activity was analyzed in db/db diabetic mice by immunofluorescence and Western blotting. **(A)** Representative immunofluorescence staining for pS6 on kidney sections. **(B-F)** Western blot analysis **(B)** and quantification of the ratio of phosphorylated to total p70 S6 ribosomal protein kinase (pS6K/tS6K), the ratio of phosphorylated to total ribosomal protein S6 (pS6/tS6), eEF2K, eEF2, and β-actin **(C-F)**. Data are expressed as mean ± SEM of 4 mice per group. Statistical analysis was performed using two-way ANOVA followed by Tukey’s or Dunnett’s *post hoc* test. Scale bar, 50 μm. # *p* < 0.05 versus db/db control. mTORC1, mammalian target of rapamycin complex 1; S6K, p70 S6 ribosomal protein kinase; S6, ribosomal protein S6; eEF2K, eukaryotic elongation factor 2 kinase; eEF2, eukaryotic elongation factor 2.

Next, we examined the activity of mTORC1 in renal tissue, which was previously associated with the protective effects of SGLT2 inhibitors by regulating fibrogenesis (20). A reduction in pS6 fluorescence intensity, indicating mTORC1 activity, was observed in renal tubules from both luseogliflozin- and glargine-treated db/db mice compared with the db/db control group (**Figure 7A**). However, Western blot analysis showed no significant changes in the pS6/tS6 ratio or the pS6K/tS6K ratio, but a significant reduction in eukaryotic elongation factor 2 kinase (eEF2K) in both treatment groups (**Figures 7B–F**). Although we cannot exclude the potential involvement of mTORC1 activity via other downstream pathways, including 4EBP1, these findings suggest that the drug-specific renoprotective effects of SGLT2 inhibitors might result from mechanisms other than the mTORC1 pathway.

### 3.4 Luseogliflozin activates renal BCKDC through dephosphorylation, in contrast to insulin glargin

**Figure 8:**
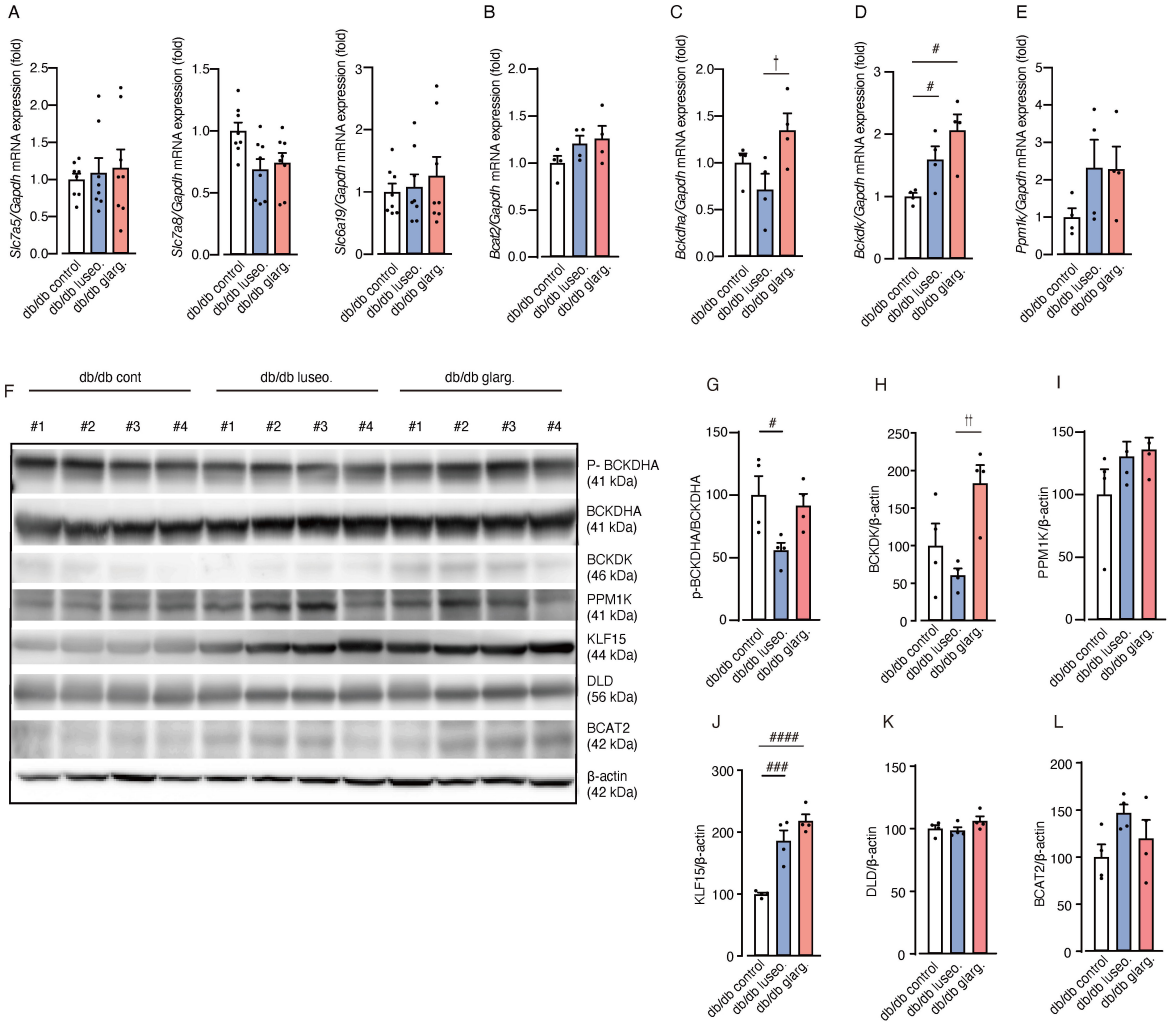
Luseogliflozin induces dephosphorylation of BCKDHA. The effects of luseogliflozin and insulin glargine treatment on the renal BCAA catabolic pathway were analyzed in db/db mice. **(A)** Relative mRNA levels of amino acid transporters (*Slc7a5, Slc7a8 and Slc6a19*) were measured by quantitative real-time PCR. **(B-E)** Expression of BCAA catabolism related genes: *Bcat2***(B)**, *Bckdha***(C)**, *Bckdk***(D)** and *Ppm1k***(E)**. **(F-L)** Western blot analysis and quantification of BCKDHA,p-BCKDHA, KLF15, BCAT2, PPM1K, BCKDK, DLD, and β-actin in kidney lysates (*n* = 4/group). Data represent the mean ± SEM of 4–8 mice per group. Statistical analysis was performed with two-way ANOVA followed by Tukey’s or Dunnett’s *post hoc* test. # *p* < 0.05 versus db/db control, †*p* < 0.05 db/db luseogliflozin versus db/db glargine. See also **Supplementary Figures S3-S6**. Slc, solute carrier family; Bcat, BCAA aminotransferase; Bckdha, BCKDHA, branched-chain ketoacid dehydrogenase E1 subunit alpha; Bckdk, BCKDK, branched-chain alpha-ketoacid dehydrogenase kinase; KLF15, Krüppel-like factor 15; BCAT2, branched chain amino acid transaminase2; PPM1K, protein phosphatase, Mg2+/Mn2+ dependent 1K; BCKDK, branched chain ketoacid dehydrogenase kinase; DLD, Dihydrolipoamide Dehydrogenase.

To evaluate the impact of luseogliflozin and glargine on renal BCAA metabolism, we analyzed the gene expression related to BCAA metabolism. No significant differences were observed between the groups in the mRNA expression of amino acid transporters (*Slc7a5*, *Slc7a8*, and *Slc6a19*) and of *Bcat2*, the key enzyme involved in the initial step of BCAA catabolism (**Figures 8A, B**).

In contrast, the gene expression of branched-chain ketoacid dehydrogenase E1 subunit alpha (*Bckdha*), a subunit of the BCKDC responsible for the second and rate-limiting step in BCAA degradation, was significantly downregulated in luseogliflozin-treated mice compared with those treated with glargine (**Figure 8C**). In addition, the mRNA expression of BCKD kinase (*Bckdk*), which inactivates BCKDC, was significantly increased in both the luseogliflozin and glargine groups compared with the control (**Figure 8D**). The expression of the *Ppm1k* gene, which encodes an activator of BCKDC, showed no significant differences between the groups (**Figure 8E**).

We then performed an analysis of protein expression. In contrast to the mRNA expression analysis, Western blotting revealed that luseogliflozin reduced the phosphorylated/inactivated form of BCKDHA (p-BCKDHA/BCKDHA), whereas glargine had no significant effect on phosphorylation (**Figures 8F, G**). BCKDK levels were also reduced in the luseogliflozin group compared with the glargine group (**Figures 8F, H**). PPM1K did not differ significantly between the groups (**Figures 8F, I**).

Krüppel-like factor 15 (KLF15), a critical transcription factor that regulates enzymes reported to increase BCAT2 and BCKDHA levels (26), showed significant increases in both the luseogliflozin and glargine groups (**Figures 8F, J**). DLD, which encodes the E3 component of the BCKDC, and BCAT2 showed no significant differences between the groups (**Figures 8F, K, L**). The expression of genes associated with glycogenesis, ketone metabolism or fatty acid metabolism, did not exhibit significant changes between the groups except for *Scd1* (**Supplementary Figure S2**).

**Figure 9:**
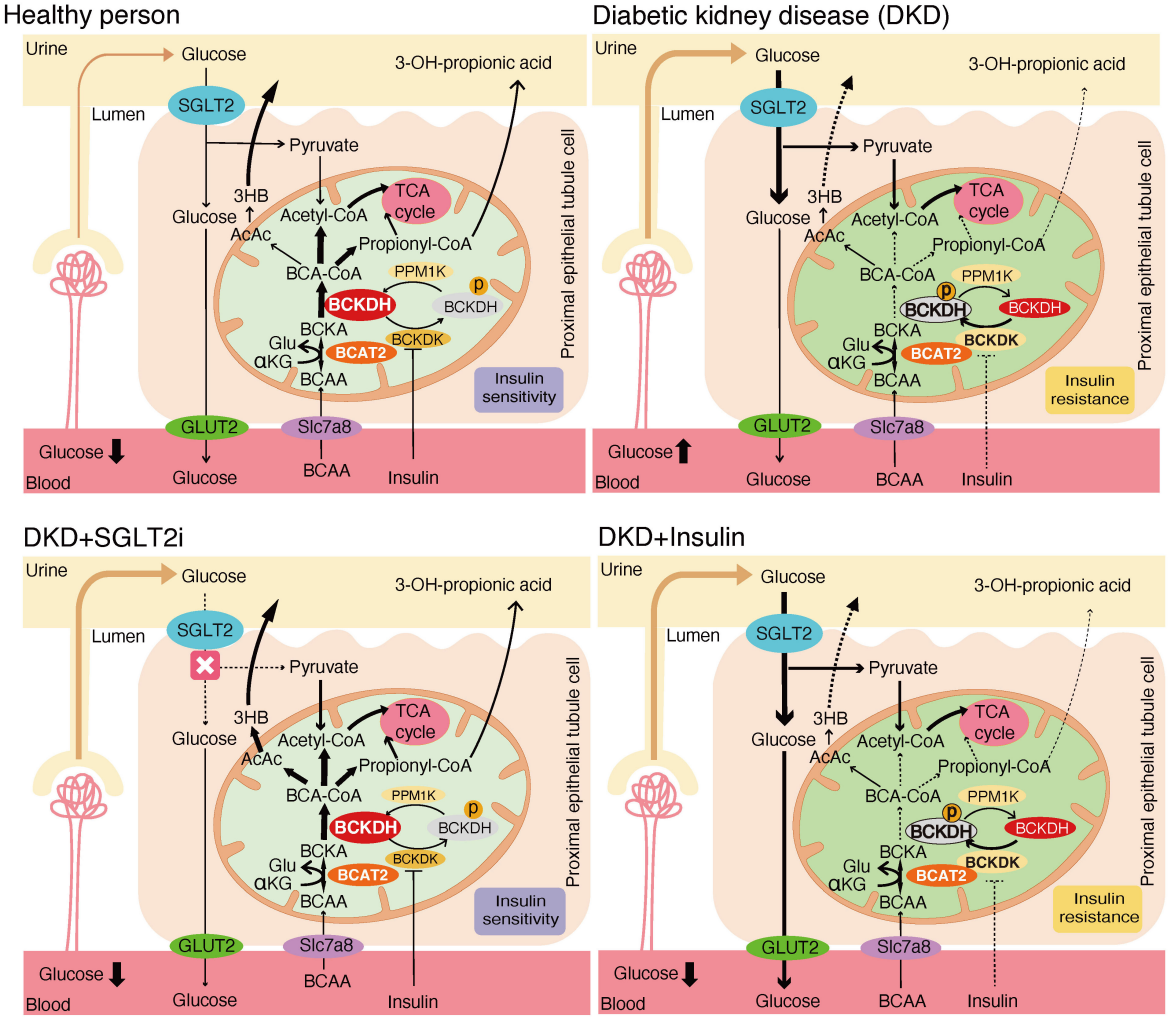
Mechanistic model of the effects of SGLT2 inhibitors and insulin treatment on renal BCAA catabolism.

These results suggest that the effects of SGLT2 inhibitors on renal BCAA catabolism are mediated by BCKDC activation through inhibition of phosphorylation, rather than transcriptional regulation. To provide an overview, **Figure 9** illustrates the renal BCAA catabolism in DKD and the potential effects of the drugs.

## 4 Discussion

In the present study, SGLT2 inhibitor treatment was associated with enhanced BCAA catabolism in humans and mice, compared with insulin treatment at comparable glycemic levels. In humans, the SGLT2 inhibitor dapagliflozin induced greater increases in urinary C5-OH carnitine, 3-hydroxybutyric acid and 3-hydroxypropionic acid, downstream degradation products of BCAA, compared with insulin. In mice, the SGLT2 inhibitor luseogliflozin decreased BCKDK and phosphorylated/inactivated form of BCKDHA and reduced glomerular mesangial expansion and the fibrosis-related gene *Col1a1* expression, along with TGFβ1 expression, compared with glargine. This study provides novel evidence that SGLT2 inhibitors enhance renal BCAA catabolism compared with insulin at comparable blood glucose levels.

Leucine, isoleucine, and valine are essential BCAAs abundant in dietary protein. Higher dietary BCAA intake has been associated with increased mitochondrial biogenesis, enhanced physical endurance, longer lifespan, and a lower risk of cardiovascular disease and all-cause mortality (27–29). However, elevated serum BCAA levels and BCAA-supplemented high-fat diets have been associated with greater insulin resistance and a higher incidence of type 2 diabetes (30, 31). Individuals with reduced insulin sensitivity or those who later developed type 2 diabetes had significantly higher plasma valine and leucine levels (32). Fasting BCAA levels during routine examinations predicted future diabetes onset in otherwise healthy, normoglycemic individuals (33). These findings suggest that BCAA utilization rate may play a crucial role in the pathogenesis of type 2 diabetes.

Sharma et al. identified 13 urinary metabolites that were significantly reduced in patients with diabetes and CKD compared with healthy controls and/or patients with diabetes without CKD, six of which were involved in BCAA catabolism (16). Interestingly, they found that among these metabolites, only 3-hydroxypropionic acid showed no significant difference between patients with diabetes with CKD and those without CKD. This finding suggests that urinary 3-hydroxypropionic acid is already reduced in the early stages of diabetes, before the onset of nephropathy. 3-hydroxypropionic acid is produced from propionyl-CoA, which is an intermediate of the catabolism of BCAAs (leucine, isoleucine, and valine) or of other compounds including methionine, threonine, and odd-chain fatty acids. Thus, the observed change in urinary 3-hydroxypropionic acid may not exclusively reflect alterations in BCAA catabolism. However, the consistent increases observed in urinary C5-OH carnitine and 3-hydroxybutyric acid, and the absence of corresponding changes in plasma metabolites also support the enhancement of BCAA catabolism in the kidney induced by SGLT2 inhibitors. We also observed a significant increase in urinary C4-carnitine, an earlier metabolite in valine catabolism, in the insulin-treated patients compared with the dapagliflozin group. Increased plasma C4-acylcarnitine has been associated with coronary artery disease (34).

Several studies have shown that SGLT2 inhibitors promote BCAA catabolism in the myocardium. In a porcine model of heart failure, empagliflozin normalized myocardial BCAA uptake and increased BCKDC activity with decreased phosphorylation, switching myocardial fuel utilization away from glucose to BCAA (14). Similarly, an untargeted metabolomics analysis in patients with type 2 diabetes mellitus and cardiovascular disease showed that empagliflozin activates BCAA catabolism (35). In contrast, few studies have addressed the effect of SGLT2 inhibitors on BCAA metabolism in the kidney, where SGLT2 is almost exclusively expressed (36). A study on this issue showed that dapagliflozin reversed the upregulation of amino acid transporters (*Slc7a8* and *Slc6a19*) and increased *Bcat1* and *Bckdha* gene expression in diabetic Akita mice, leading to the inhibition of mTORC1 in the proximal tubule and the prevention of renal fibrogenesis when compared with untreated controls (20). However, it remained unclear whether these changes were induced by glucose-lowering or by a drug-specific mechanism of SGLT2 inhibitors.

In *in vivo* experiments with db/db diabetic mice, we found that luseogliflozin reduced renal injury compared with glargine. Luseogliflozin significantly reduced the phosphorylation ratio of BCKDHA, and decreased BCKDK levels compared with glargine. We did not detect differences in mTOR signaling between luseogliflozin- and glargine-treated db/db mouse groups. Together with the observed differences in urinary BCAA breakdown products between patients treated with dapagliflozin and those treated with insulin alone, these results suggest that SGLT2 inhibitors promote BCAA catabolism and exert a renoprotective effect.

The mechanism by which SGLT2 inhibitors enhance intracellular BCAA catabolism requires further investigation. Insulin inhibits BCKDK, which inactivates BCKDHA. Therefore, the reduction in BCKDK and decreased phosphorylation/inactivation of BCKDHA induced by luseogliflozin could be explained by improved insulin sensitivity. A very recent study demonstrated that enhancement of BCAA catabolism through inhibition of BCKDK improved kidney function, supporting the potential relevance of this pathway (37). Another possibility is that SGLT2 inhibition promotes BCAA catabolism as part of metabolic adaptations to energy and water shortages, leading to the generation of organic osmolytes (38). Urea, a typical organic osmolyte, is produced from ammonia via the oxidative deamination of glutamate. We observed an increasing trend in plasma glutamate levels in dapagliflozin-treated patients. The catabolism of BCAA provides nitrogen for glutamate synthesis. Notably, BCKDK knockout mice, which exhibit increased systemic BCAA oxidation, were reported to have increased plasma glutamate levels (15), suggesting a potential link between BCAA catabolism and blood glutamate levels.

SGLT2 inhibitors are known to induce a mild increase in blood ketone bodies, although the detailed mechanism remains incompletely understood. Recently, it has been reported that renal proximal tubular cells under fasting conditions express 3-hydroxymethylglutaryl-CoA synthase (HMGCS2), the rate-limiting enzyme of ketogenesis, which is normally exclusive to hepatocytes (39). However, we found no significant differences in renal HMGCS2 mRNA expression between the mouse treatment groups. In contrast, we observed an increase in urinary 3-hydroxybutyric acid in the dapagliflozin-treated group compared with the insulin-treated group. Amino acid catabolism, particularly of leucine, is another known source of ketone bodies (40). Recent human data demonstrated that dapagliflozin treatment increased whole-body fat oxidation and tended to increase protein oxidation (41). SGLT2 inhibitors are associated with an increased risk of euglycemic diabetic ketoacidosis in both type 1 and type 2 diabetes (42). Based on these findings, we propose that a shift in fuel metabolism, including enhanced BCAA catabolism, may partially contribute to the tendency of SGLT2 inhibitors to elevate systemic ketone levels. Further studies are needed to explore the effect of SGLT2 inhibitors on inter-organ coupling of fuel utilization, ketogenesis, and BCAA catabolism.

This study has several limitations. First, only 13 patients completed the study. Some measurements showed relatively large ranges. Because no prior reports examined differences in urinary metabolite changes between the SGLT2 inhibitor- and insulin-treated groups, we selected a sample size based on feasibility, aiming to detect clinically relevant differences in exploratory analyses. Therefore, the following null hypothesis was tested: There is no difference in urinary metabolite changes between dapagliflozin and insulin dose-up treatments. A prospective controlled study was designed, and a statistically significant difference in changes in urinary 3-hydroxypropionic acid between the two treatment groups was observed at an alpha level of 0.05, leading to the rejection of the null hypothesis. For reference, in a *post hoc* analysis, the statistical power for the observed difference was 0.65 (sample size was set at 7), while a statistical power of 0.80 or higher is typically preferred. However, given that this was an exploratory phase study, the obtained statistical power of 0.65 was considered acceptable to provide a basis for designing a larger-scale study in the future.

Second, we used different SGLT2 inhibitors in this study. Although the cardio-renal benefits of SGLT2 inhibitors are considered to be a class effect, the possibility of a drug-specific effect on renal BCAA metabolism cannot be excluded. Luseogliflozin was chosen for the experimental models due to its superior selectivity for SGLT2 over SGLT1, which was particularly relevant for evaluating the effects of SGLT2 inhibition in this context. For the clinical study, dapagliflozin was selected because, at the time, it was the only SGLT2 inhibitor approved in Japan for the treatment of chronic kidney disease. Its use was therefore considered promising for patient benefit.

Our results demonstrate that SGLT2 inhibitors—but not insulin—enhance BCAA catabolism by activating BCKDHA in the kidney. The concomitant improvement in renal histology suggests the therapeutic relevance of BCAA catabolism in the context of DKD.

## Data Availability

The datasets generated during the current study are available from the corresponding author on reasonable request.

## Glossary

ACC: acetyl CoA carboxylase
ACS: acyl-CoA synthetase
ALT: alanine aminotransferase
AST: aspartate aminotransferase
BCA-CoA: branched-chain acyl-CoA
BCAA: branched chain amino acid
BCAT2: branched-chain aminotransferase 2
BCKA: branched-chain α-ketoacids
BCKDC: branched-chain α-ketoacid dehydrogenase complex
BCKDHA: branched chain ketoacid dehydrogenase E1 subunit alpha
BCKDK: branched-chain α-ketoacid dehydrogenase kinase
BUN: blood urea nitrogen
CPR: C-peptide immunoreactivity
CPT1A: carnitine palmitoyltransferase 1A
Col1a1: collagen type I alpha 1 chain
Cre: creatinine
DKD: diabetic kidney disease
DLD: dihydrolipoyl dehydrogenase
ESRD: end-stage renal disease
FPG: fasting plasma glucose
G6pc1: glucose-6-phosphatase catalytic subunit 1
Glu: glutamate
HMGCS2: 3-hydroxy-3-methylglutaryl-CoA synthase 2
KLF15: kr̈ppel-like factor 15
LFABP: liver-type fatty acid-binding protein
MSDKD: metabolite score for diabetic kidney disease
PAS: periodic acid-Schiff
PPARa: peroxisome proliferator-activated receptor α
Ppm1k: protein phosphatase, Mg2+/Mn2+ dependent 1K
SCOT: succinyl-CoA:3-ketoacid CoA transferase
SGLT2i: sodium-glucose co-transporter 2 inhibitors
Slc: solute carrier
Srebp1: sterol regulatory element-binding protein 1
TCA: tricarboxylic acid
TG: triglyceride
UA: uric acid
eEF2: eukaryotic elongation factor 2
eEF2K: eukaryotic elongation factor 2 kinase
mTORC1: mammalian target of rapamycin complex 1
pS6: phosphorylated S6 ribosomal Protein
pS6K: phosphorylated p70 S6 ribosomal protein kinase
tS6: total S6 ribosomal Protein.
