# Supplementary material for: Sodium-glucose cotransporter 2 inhibitors—but not insulin—enhance renal branched-chain amino acid catabolism
Source: Front Endocrinol (Lausanne). 2025 Nov 7;16:1706838. doi: 10.3389/fendo.2025.1706838 (PMC12634333; doi:10.3389/fendo.2025.1706838)
Supplement: Supplementary file 1 [file DataSheet1.docx]

Supplementary Material

# Supplementary Figures and Tables

##
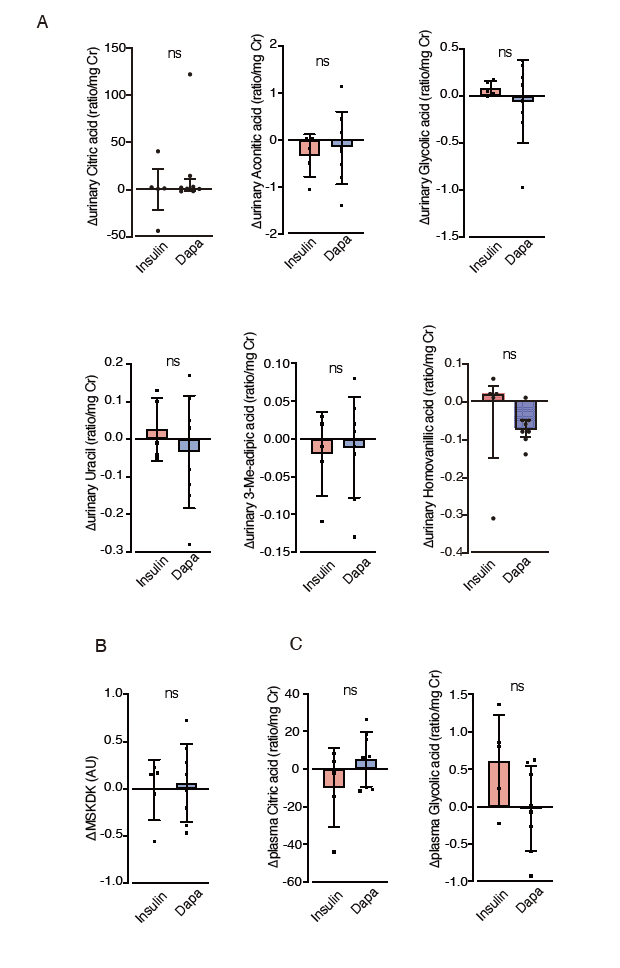
Supplementary Figures

**Supplementary Figure 1.　Changes (Δ) in urinary and plasma metabolites after 12 weeks of dapagliflozin or insulin treatment in patients with diabetes.** (A) Changes (Δ) in other detected urinary metabolites. (B) Changes (Δ) in the metabolite score for diabetic kidney disease MSDKD, which was calculated from both the metabolites shown in Figure 2 and those in Figure S1. (C) Changes in other detected plasma metabolites.Data are presented as mean ± SD for normally distributed variables or as median with interquartile range for non-normally distributed variables. Statistical analysis was performed using unpaired Student’s t-test or the Mann–Whitney test, as appropriate.


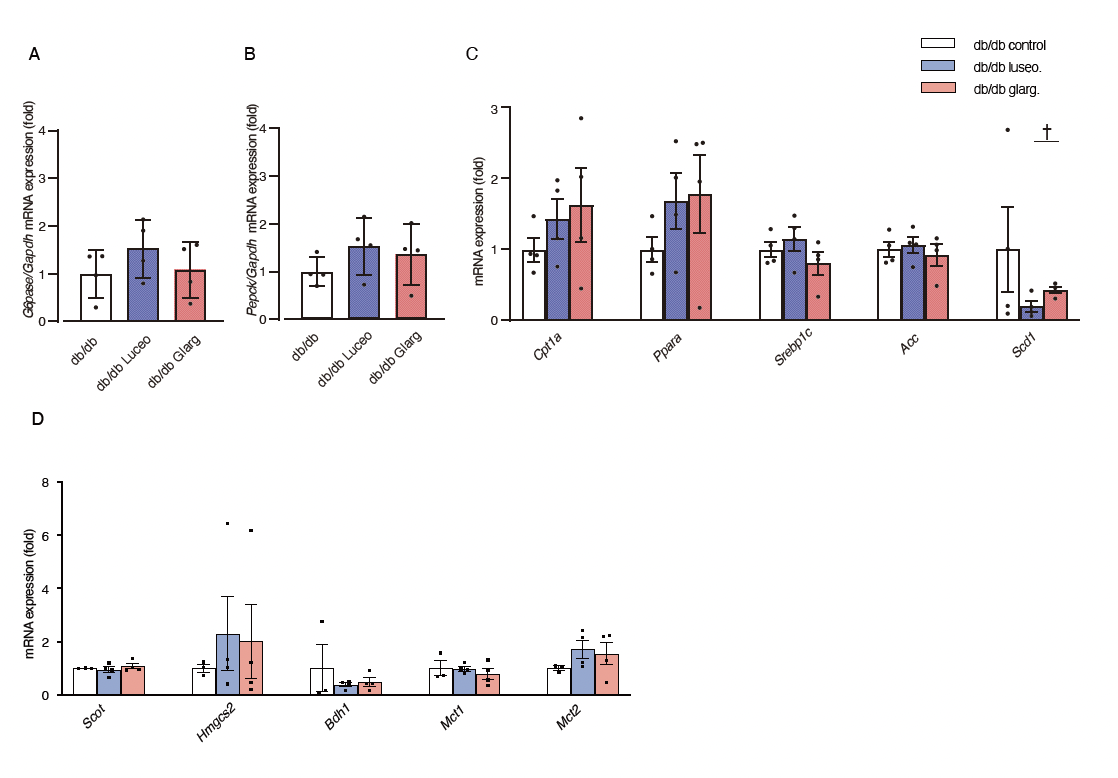


**Supplementary Figure 2. Expression of genes associated with glycogenesis, ketone metabolism, and fatty acid metabolism in the luseogliflozin- and glargine-treated mice.** Expression of genes associated with glycogenesis (A:G6pc1, B:Pepck), ketone metabolism (C), and fatty acid metabolism (D). mRNA expression was normalized to the level of Gapdh. Data represent the mean ± SD of 4 mice per group. Statistical analysis was performed using two-way analysis of variance (ANOVA) followed by Tukey's or Dunnett's post hoc test. †p < 0.05 db/db luseogliflozin versus db/db glargine.


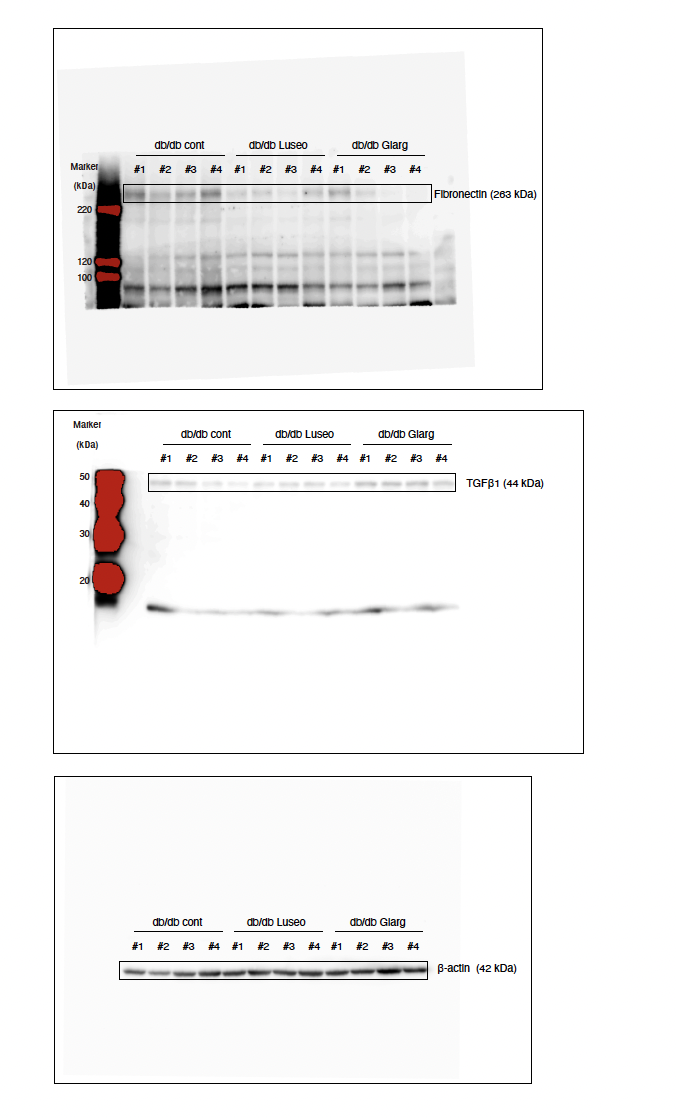


**Supplementary Figure 3. Full blot pictures related to Figure 6.**


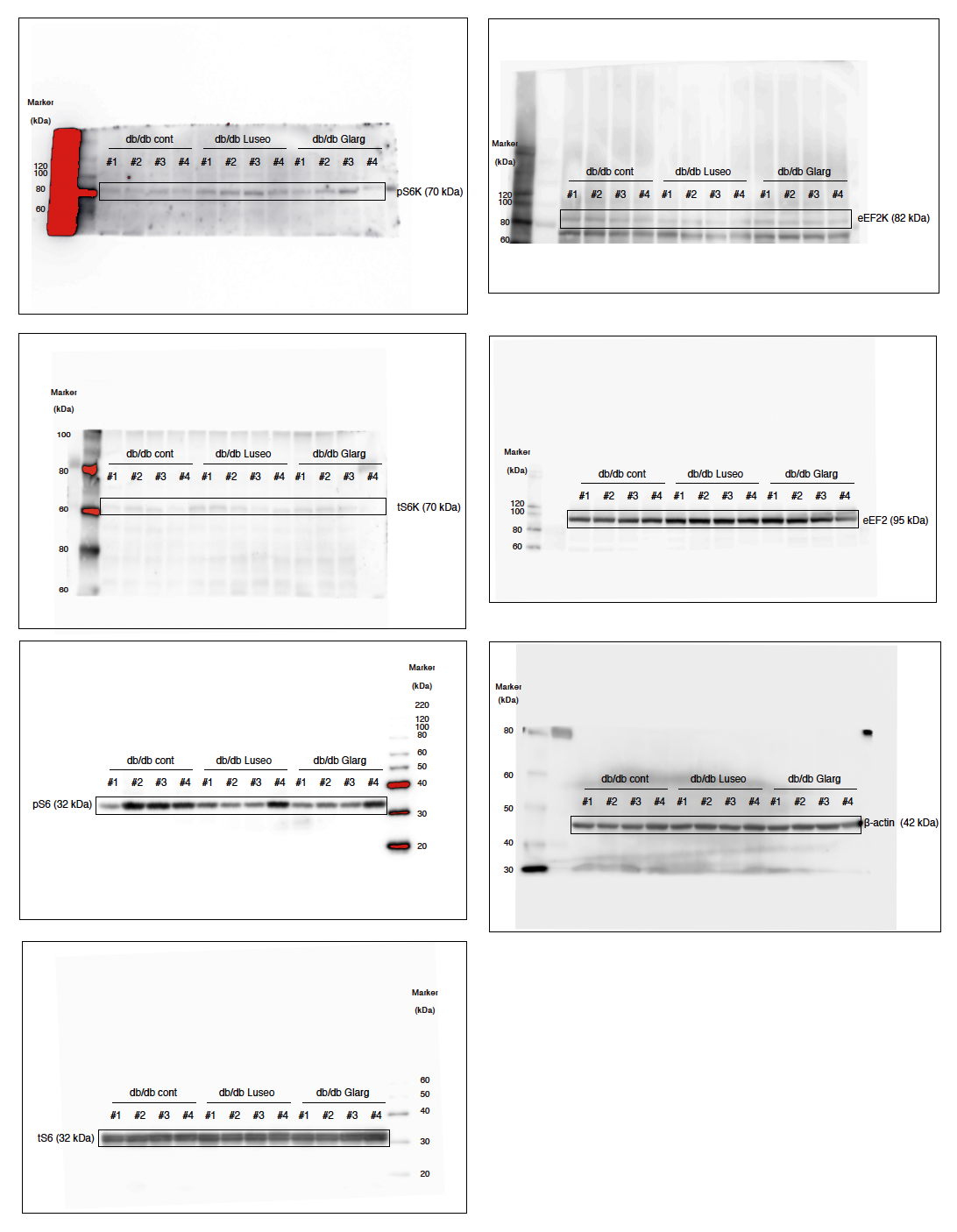


**Supplementary Figure 4. Full blot pictures related to Figure 7B**


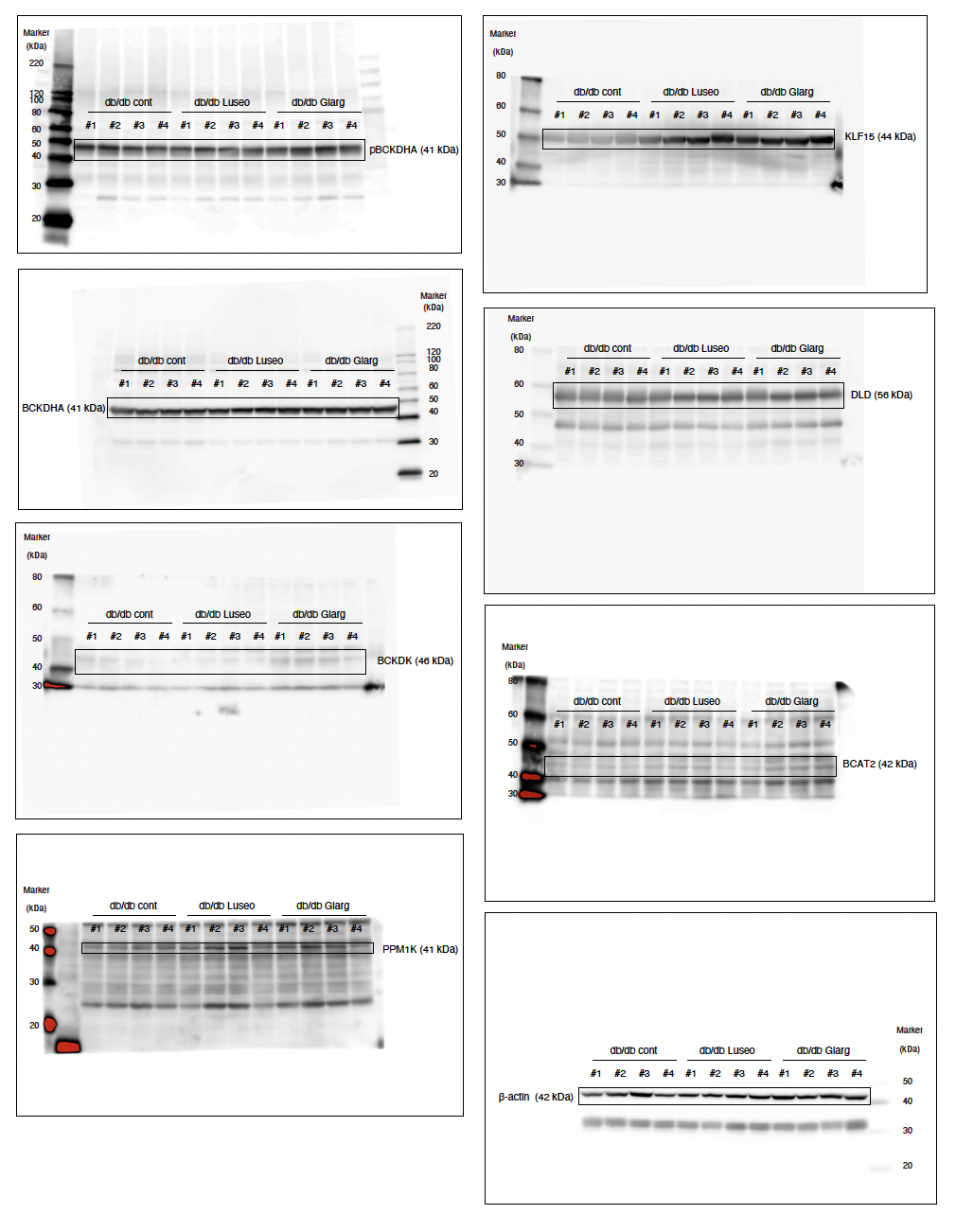


**Supplementary Figure 5. Full blot pictures related to Figure 8F**

## Supplementary Tables

**Supplementary Table S1. Primer sequences used for qPCR analysis of specific genes**

| Gene | Forward primer (5’-3’) | Reverse primer (5’-3’) | Product size (bp) |
| --- | --- | --- | --- |
| *Col1a1* | GCGGTTCAGGTCCAATGGGT | GTTCCAGGCAATCCACGAGC | 139 |
| *Col1a2* | CCGTGCTTCTCAGAACATCA | GAGCAGCCATCGACTAGGAC | 168 |
| *Tgfb* | GAGCCCGAAGCGGACTACTA | CACTGCTTCCCGAATGTCTGA | 188 |
| *Tnfa* | CCACCACGCTCTTCTGTCTA | AGGGTCTGGGCCATAGAACT | 103 |
| *Il1b* | TGTGAAATGCCACCTTTTGA | GGTCAAAGGTTTGGAAGCAG | 94 |
| *Slc7a5* | CCTACGGAGGATGGAACTATCT | TGACAATGGGCAAGGAGATG | 99 |
| *Slc7a8* | GGTGGCTGGAACTTCCTTAAT | CAGTGGGATGGAGATGAAGATG | 90 |
| *Slc6a19* | GGAGTGTGCTGTATGTGTGTAT | TCAAGCCACGGATGAGAAAG | 114 |
| *Bcat2* | CCATAGTTCCCCCCCAACTT | CGGACCCTTCATTCGTCAGA | 67 |
| *Bckdha* | CCAGGGTTGGTGGGATGAG | GGCTTCCATGACCTTCTTTCG | 70 |
| *Bckdk* | GATCCGAATGCTGGCTACTCA | GCCAACAAAATCAGGCTTGTC | 61 |
| *Ppm1k* | AACTGTCCTGACCTTGGCCT | CCGTGGTGCACTTTTCCATCT | 87 |
| *G6pase* | AACGCCTTCTATGTCCTCTTTC | GTTGCTGTAGTAGTCGGTGTCC | 70 |
| *Peepck* | TGGAAGGTCGAATGTGTGGG | AGCCCTTAAGTTGCCTTGGG | 69 |
| *Cpt1a* | CCAGGCTACAGTGGGACATT | GAACTTGCCCATGTCCTTGT | 209 |
| *Ppara* | CGGGATGTCACACAATGCAA | ACAAAAGGCGGGTTGTTGCT | 228 |
| *Srebp1* | GATCAAAGAGGAGCCAGTGC | TAGATGGTGGCTGCTGAGTG | 191 |
| *Acc* | CCAGGCCATGTTGAGACGCT | ATCACAGAGCGGACGCCATC | 132 |
| *Scd1* | AGTGCCGCGCATCTCTATG | AAGGGGAAGGTGTGGTGGT | 122 |
| *Scot* | TGGCCAACTGGATGATACCTGG | TCCATGGTGACCACCACTTTGG | 97 |
| *Hmgcs2* | TCAACTCCCTGTGCCTGACA | CAATGATGGTCTCGGTGCCC | 102 |
| *Bdh1* | TCGTGTCCTCTAGACAGCCC | CCGAGGCAGAGAGAGCAATC | 69 |
| *Mct1* | GCCCCTTGAAACCTATGGCT | TGTGGTTGACATCCAGTCCG | 61 |
| *Mct2* | TGGCATCACAAGCACCTATGA | TACGATGTAGATAATGGTTGCTGC | 52 |
| *Gapdh* | CCATCACTGCCACCCAGAAG | GATGCAGGGATGATGTTC | 91 |

**Supplementary Table S2. Primer sequences used for qPCR analysis of specific genes**

| Antibodies | | |
| --- | --- | --- |
| Rabbit anti-Fibronectin | Proteintech Group | Cat#15613-1-AP; RRID: AB_2105691 |
| Rabbit anti-TGF1b | Abcam | Cat#AB215715; RRID: AB_2893156 |
| Rabbit anti-pS6(Ser235/236) | Cell Signaling Technology | Cat# 4858S; RRID: AB_916156 |
| Rabbit anti-tS6 | Cell Signaling Technology | Cat# 2217S; RRID: AB_331355 |
| Rabbit anti-Phospho-p70 S6 Kinase(Thr389) | Cell Signaling Technology | Cat# 9205; RRID:AB330944 |
| Rabbit anti-p70 S6 Kinase | Gene Yex | Cat# GTX107562 |
| Rabbit anti-EEF2 | Proteintech Group | Cat# 20107-1-AP; RRID:AB_10950401 |
| Rabbit anti-eEF2K | Proteintech Group | Cat# 13510-1-AP; RRID: AB_2277761 |
| Rabbit anti-KLF 15 | Proteintech Group | Cat# 13749-1-AP |
| Rabbit anti-DLD | Proteintech Group | Cat# 16431-1-AP |
| Rabbit anti-BCAT2 | Proteintech Group | Cat# 16417-1-AP; RRID:AB_10792411 |
| Rabbit anti-p-BCKDHA (S293) | Abcam | Cat# ab200577 |
| Rabbit anti-BCKDHA | Cell Signaling Technology | Cat# 90198S; RRID: AB_2800155 |
| Rabbit anti-PPM1K | Proteintech Group | Cat# 14573-1-AP; RRID: AB_2252953 |
| Rabbit anti-BCKDK | Proteintech Group | Cat# 15718-1-AP; RRID: AB_2878174 |
| Mouse anti-β-actin | Cell Signaling Technology | Cat# 4970S; RRID:AB_222317 |
| Anti-mouse IgG, HRP-linked Antibody | Cell Signaling Technology | Cat# 7076S RRID:AB_330924 |
| Anti-rabbit IgG, HRP-linked Antibody | Cell Signaling Technology | Cat# 7074S RRID:AB_2099233 |
| Cystatin C antibody | Abcam | Cat#ab109508;RRID:AB_10888303 |
